# A Novel Smartphone App for Self-Monitoring of Neonatal Jaundice Among Postpartum Mothers: Qualitative Research Study

**DOI:** 10.2196/53291

**Published:** 2023-12-22

**Authors:** Aminath Shiwaza Moosa, Alvin Jia Hao Ngeow, Yuhan Yang, Zhimin Poon, Ding Xuan Ng, Eileen Koh Yi Ling, Ngiap Chuan Tan

**Affiliations:** 1 SingHealth Polyclinics, Primary Care Research Institute, Singapore, Singapore; 2 Family Medicine Academic Clinical Programme, SingHealth Duke-NUS Academic Medical Centre, Singapore, Singapore; 3 Department of Neonatology, Singapore General Hospital, Singapore, Singapore; 4 Paediatrics Academic Clinical Programme, SingHealth Duke-NUS Academic Medical Centre, Singapore, Singapore; 5 Department of Paediatrics, Yong Loo Ling School of Medicine, National University of Singapore, Singapore, Singapore; 6 Lee Kong Chian School of Medicine, Nanyang Technological University, Singapore, Singapore

**Keywords:** neonatal jaundice screening, mobile health, usability, primary care, neonatology, neonatal, care, self-monitoring, monitoring, smartphone application, application, app, mHealth app, users, screening, postpartum, postpartum mother, usability, utility, data management, applicability, teleconsultation, neonatal jaundice, mHealth

## Abstract

**Background:**

Neonatal jaundice (NNJ) or hyperbilirubinemia is a ubiquitous condition in newborn infants. Currently, the transcutaneous bilirubinometer is used to screen for NNJ in health care facilities, where neonates need to be physically present (ie, a centralized model of care for NNJ screening). Mobile health (mHealth) apps present a low-cost, home-based, and noninvasive system that could facilitate self-monitoring of NNJ and could allow mothers the convenience of screening for NNJ remotely. However, end users’ acceptability of such mHealth apps is of fundamental importance before the incorporation of such apps into clinical practice.

**Objective:**

The study aimed to explore the perception of postpartum mothers toward self-monitoring of NNJ using a novel mHealth app.

**Methods:**

Mothers attending video consultations for early postpartum care at 2 Singapore primary care clinics watched an instructional video for a hyperbilirubinemia-screening mHealth app (HSMA). An independent researcher used a semistructured topic guide to conduct in-depth interviews with 25 mothers, assessing their views on HSMAs. All interviews were audio recorded, transcribed verbatim, and checked for accuracy before data analysis. Two researchers independently analyzed the transcripts via thematic analysis. Data were managed using NVivo qualitative data management software.

**Results:**

The identified themes were grouped under perceived usability and utility. Mothers valued the convenience and utility of HSMAs for remote monitoring of NNJ. They appreciated the objectivity the app readings provided compared to visual inspection. However, they perceived that the app’s applicability would be restricted to severe jaundice, were concerned about its accuracy and restriction to the English language, and lacked confidence in using it. Nevertheless, they were willing to use it once its accuracy was proven and when they received adequate guidance from health care professionals. They also suggested including an action plan for the measured readings and clinical signs within the app. Mothers proposed pairing teleconsultations with HSMAs to boost their confidence and enhance adoption.

**Conclusions:**

Mothers were receptive to using HSMAs but had concerns. Multiple languages, proof of accuracy, and resources to guide users should be incorporated into the app in the next phase to increase its successful adoption. Complementing such apps with a teleconsultation service presents a plausible and pragmatic NNJ care delivery model in general practice.

## Introduction

Neonatal jaundice (NNJ) is common in newborn infants. It is secondary to elevated total serum bilirubin or hyperbilirubinemia [1]. The prevalence of NNJ is 60% and 80% in term and preterm neonates, respectively [2]. Physiological jaundice accounts for most cases of neonatal hyperbilirubinemia [1]. It is usually mild and self-limiting [1]. However, in severe hyperbilirubinemia, unconjugated bilirubin may cross the blood-brain barrier and lead to neurological deficits [1]. The incidences of acute and chronic bilirubin encephalopathy are approximately 1 in 10,000 live births and 1 in 50,000 to 100,000 live births, respectively [1]. Bilirubin-induced encephalopathy can result in cerebral palsy, with an incidence of 0.57 in 100,000 live births [3,4]. As such, infants with NNJ require close surveillance, and immediate medical attention is warranted in severe hyperbilirubinemia.

Total serum bilirubin measurement remains the gold standard for ascertaining NNJ [5]. However, this method involves invasive blood sampling of the neonate, whereas the existing local standard of care requires NNJ monitoring using the transcutaneous bilirubinometer. The latter is a specialized equipment only available in health care facilities. This creates a necessity for parents to bring their babies to clinics for jaundice checks [5], resulting in the incurrence of travel costs and time away from work by parents, as well as the potential risk of exposure to infectious pathogens at health care facilities [6,7].

Monitoring for NNJ through smartphone apps presents a low-cost, home-based, and noninvasive system that could facilitate self-monitoring of NNJ and reduce unnecessary exposure of the child to health care facilities. Such smartphone apps have been developed in China, the Unites States, and Norway [8-10]. Currently, these apps are in the research phase with promising results. Rong et al [8], Taylor et al [9], and Aune et al [10] reported good overall associations between the app-based bilirubin estimates and total serum bilirubin, with Pearson correlation coefficients (*r*) of 0.788, 0.91, and 0.84, respectively. Thus, the use of such smartphone apps offers a convenient and viable alternative for laypersons such as postpartum mothers for the purpose of NNJ screening.

At present, such mobile health (mHealth)–based, NNJ-monitoring tools are novel to the local multiethnic parents in cosmopolitan Singapore. Little is known about parental levels of awareness and acceptability. Scaling up the use of such apps is only possible if the end users are receptive to their use [11]. Engaging end users in the development and design of mHealth technologies eases the adoption and accessibility of such technologies [12]. Thus, this study aimed to explore the views of mothers toward a novel mHealth app for their self-monitoring of NNJ based on their perceived usefulness and concerns. Findings and feedback from these end users can be used to enhance the features of the mHealth app, which will improve its usability and utility to monitor for NNJ remotely.

## Methods

### Study Design

Qualitative research methodology was used to garner the perceptions of mothers on the usefulness of NNJ-monitoring or hyperbilirubinemia-screening mHealth apps (HSMAs). Biliscan (Shenzhen Beishen Healthcare Technology Co) is one such app being validated at a local tertiary hospital [13]. The usefulness of an app is defined as a combination of its usability and utility [14]; usability is the ease of use of the user interface, and utility refers to the functionality of the app and its value to users [14].

This study is reported according to the COREQ (Consolidated Criteria for Reporting Qualitative Research) checklist (Checklist 1).

### Study Setting

The study sites were 2 public primary care clinics (polyclinics) in the Bukit Merah (BM) and Sengkang (SK) estates located in southern and eastern Singapore, respectively. The latter is populated with young families and manages over 1000 patients daily during office hours. The primary care physicians and nurses in these 2 polyclinics provide comprehensive primary care to mothers and children, including screening and monitoring neonates for NNJ.

### Research Team

The research team included 2 female primary care physicians (ASM and ZP), 1 male primary care physician (NCT), 1 male neonatologist (AJHN), and 1 female student (YY). ASM, AJHN, and ZP are trained in qualitative and quantitative research. NCT is a trainer and experienced researcher in both qualitative and quantitative research.

### Study Population and Recruitment

Mothers aged 21 years and older, within 4 weeks of their postpartum period, and who spoke English were eligible for the study. Primary care physicians and nurses at the study site helped identify these mothers when they attended the clinic for their child’s NNJ follow-up. Mothers were recruited from December 2021 to August 2022.

### Ethical Considerations

The study was approved by the SingHealth Centralised Institutional Review Board (CIRB 2021/2732). The research was conducted according to International Conference on Harmonization guidelines for Good Clinical Practice. Clinical research coordinators approached potential participants face-to-face and informed them of the study purpose before they signed informed written consent. Each mother was assigned a unique study identification number to deidentify them and ensure confidentiality. They were remunerated with an SGD $20 (~US $15) grocery voucher for their time and contribution to the study.

### Study Instruments and Data Collection

#### Demographic Characteristics Survey

A web-based questionnaire was designed to collect the mothers’ demographic characteristics. The electronic survey form was hosted on FormSG, a secure, officially approved web-based platform available to the public in Singapore [15]. The questionnaire collected demographic and selected clinical formation from the mothers on their age, ethnicity, marital status, educational level, housing type, financial status, delivery date, and parity at recruitment.

#### Instructional Video on NNJ Screening Using an mHealth App

The instructional video *Jaundice Screening Using a Smartphone* [16] was created by AJHN for the purpose of patient education and consent taking as part of an earlier validation study on Biliscan [13,17]; it shows an HSMA being used for remote monitoring of NNJ. The use of HSMAs is described in detail by Alvin et al [17]. In brief, it involves placing a color calibration card with a central aperture on the baby’s sternum, aligning the app’s photograph frame with the color card, and taking a picture. After processing the image and applying a machine learning regression algorithm, bilirubin levels are estimated and displayed on the phone screen. The video was uploaded on the institute’s official YouTube channel [16]. Mothers were invited to watch this video using a QR code before their in-depth interviews.

#### Topic Guide and In-Depth Interviews

A semistructured topic guide was developed based on a literature review and discussion with team members. The questions inquired about the mother’s perceived utility and usability of HSMAs to monitor their child for NNJ. The topic guide was pilot-tested and iteratively modified based on data that emerged during both pilot and subsequent interviews.

An independent researcher (ASM, ZP, or NCT) used the topic guide to interview individual mothers over the phone. The interviewer was in the primary care clinic while the mother was at home. All mothers were unaccompanied during the interview. All interviews were audio recorded and each interview lasted 25-45 minutes. The study team recorded field notes during each interview.

### Data Analysis

Professionals transcribed the interviews, and the content of the audio recordings was audited for accuracy. Next, 2 researchers (ASM and ZP) independently coded the initial 3 transcripts to generate an initial coding frame. Based on the initial coding frame, 2 researchers (ASM and YY) coded and indexed subsequent transcripts using NVivo qualitative data management software (Lumivero). The researchers regularly discussed the qualitative data and their coding.

The researchers analyzed and grouped the codes to identify emerging themes. Any disagreements were resolved with discussion and consensus. Subsequently, the research team reviewed and revised the results to finalize the themes. Representative quotes were selected to illustrate the results. Regular iterations after each interview and referral to field notes allowed ASM to reflect on the findings and to assess for idea saturation [18]. No new idea emerged after the 23rd interview. Two more interviews were conducted before a mutually agreed decision was reached to stop further qualitative data collection.

## Results

### Participants

A total of 56 postpartum mothers were approached; 30 consented to participate in the study (a response rate of 54%), and 25 mothers completed the in-depth interviews. A total of 5 mothers were lost to follow-up: 2 after giving consent and 3 after watching the video. Mothers who declined to participate cited time constraints, a lack of interest, or other commitments that prevented them from participating in the study. All mothers completed the preconsultation questionnaire.

### Participant Characteristics

Table 1 reports the sociodemographic characteristics of the 30 mothers in the main study. Most were aged 30-34 years (15/30, 50%), were of Chinese ethnicity (21/30, 70%), obtained university or posttertiary education (26/30, 87%), lived in Housing and Development Board (Singapore’s public housing) 4- to 5-room flats (20/30, 67%), and did not receive medical subsidy (29/30, 97%). More than half (17/30, 57%) of the mothers underwent normal vaginal delivery. Around two-thirds (19/30, 63%) were first-time mothers.

**Table 1.** Demographic characteristics of the mothers.

Characteristics		Mothers (n=30), n (%)
**Age group (y)**		
	20-24	1 (3)
	25-29	5 (17)
	30-34	15 (50)
	35-39	9 (30)
**Ethnicity**		
	Chinese	21 (70)
	Malay	1 (3)
	Indian	5 (17)
	Others	3 (10)
**Highest education qualification**		
	Secondary	1 (3)
	A-level or diploma (ITE^a^, polytechnic, or private school)	3 (10)
	University or posttertiary	26 (87)
**Housing type**		
	Rental room or apartment	3 (10)
	HDB^b^ 1- to 3-room flat	5 (17)
	HDB 4- to 5-room flat	20 (67)
	Masonite or executive condominium	2 (7)
**Type of delivery**		
	Assisted delivery	2 (7)
	Caesarean section	11 (37)
	Normal vaginal delivery	17 (57)
**Number of children**		
	1	19 (63)
	2	10 (33)
	3 or more	1 (3)
**Current child feeding method**		
	Breastfeeding only	4 (13)
	Formula feeding only	1 (3)
	Mixed feeding (breastfeeding and formula feeding)	25 (83)
**Breastfeeding difficulty**		
	No	12 (40)
	Yes	18 (60)
**Medical subsidy status**		
	No	29 (97)
	Yes	1 (3)

a ITE: Institute of Technical Education.

b Housing and Development Board (Singapore’s public housing).

### Main Findings

Findings regarding the mothers’ perceived usefulness were grouped into usability and utility. The themes, their descriptions, and subthemes are listed in Table 2.

**Table 2.** Themes, their descriptions, and subthemes on mother’s perceived usefulness of hyperbilirubinemia-screening mobile health apps.

Themes	Descriptions	Subthemes
Utility	Functionality of the app and the value to users	Preference and convenience to check jaundice level at home Provides objectivity compared to visual inspection Limited applicability for severe jaundice Accuracy and reliability of the app
Usability	Ease of use of the app’s user interface and improving the ease of use	Easy to use Instructions restricted to the English language Higher trust in health care professionals Guidance for app use and the management of jaundice Incorporation into teleconsultations

### Utility

All mothers were receptive to using HSMAs because of their convenience and quantitative measurement without the need to access health care facilities. However, they were concerned about the app’s accuracy, leading to their lack of confidence in using it.

#### Preference and Convenience to Check Jaundice Levels at Home

Most mothers perceived the benefits of using HSMAs, which allowed them to monitor their children for NNJ daily in the comfort of their homes instead of needing multiple visits to a clinic or hospital. They were concerned about the risk of infection outdoors. They felt reassured to know their child’s NNJ status before the in-person review by a health care professional.

*I’m sure from time to time the mother will be curious if the numbers are going down. For paranoid mothers like me, I think it would be good if I can monitor it on my own on day-to-day basis instead of going to the polyclinic.* [SK05; 36-year-old Chinese mother of 2]

*As a small kid, you know it’s very troublesome to visit a clinic…[due to] the viruses and all outside because he’s still so young. He is a baby, so it’s not that good to make him travel around. Usually, we don’t let the child travel until he’s two months or three months [and] completed [the vaccination].* [BM06; 39-year-old Indian mother of 2]

Mothers also preferred not undergoing blood sampling for NNJ assessment, as they perceived it to be painful for their child. They recognized the use of HSMAs as a less traumatic alternative to the invasive blood test.

*I went to poly four times to do the jaundice check. And every time we went through the blood test, it pains me to see my child being pricked by the needle. I think this will be something that I will definitely use.* [SK03; 38-year-old Javanese mother of 2]

#### Provides Objective Assessment Compared to Visual Inspection

Mothers appreciated the quantitative evaluation of NNJ by HSMAs. They were more assured of the numerical measurement of jaundice than subjective visual inspection of the skin color.

*…when it comes to jaundice, we always try to see baby yellow or not, eyes yellow or not. With such a device, at least it is more certain for us to do the first cut of assessment.* [SK03; 38-year-old Javanese mother of 2]

*I think it’s quite useful, at least we have some idea instead of just pressing his skin to know his level. I am quite a statistic person. If I have numbers, I will be more reassured that the jaundice is coming down.* [SK10; 38-year-old Chinese mother of 1]

#### Limited Applicability for Severe Jaundice

Mothers were unsure of the use of HSMAs for severe NNJ, as they considered that it would warrant a clinic visit for a laboratory-based serum bilirubin measurement.

*…[in severe jaundice] we know already that we will have an appointment and then this appointment is set every 2 days because the level was still quite high up on that day [because of this] I don’t think I will use the app anyway. I will be seeing a doctor, so there’s no point for me to install the app.* [BM10; 38-year-old Chinese mother of 1]

Mothers would select in-person clinic visits to check bilirubin levels for severe NNJ. They were worried about undue delay for their child to receive the required treatment, which might result in harm.

*…if the jaundice is not that serious, I guess I am okay to check at home with this mobile app but if baby has very serious jaundice, I would still prefer to come down to polyclinic.* [SK01; 38-year-old Chinese mother of 1]

*…maybe it [jaundice level] increasing and we are not able to notify the help of doctors, it will be very painful for my baby.* [SK08; 31-year-old Indian mother of 1]

#### Accuracy and Reliability of the App

Many mothers were uncertain about the accuracy and reliability of the HSMA. They raised concerns that the impact of different camera quality and lighting on its measurements.

*If there’s a way to cite like how accurate the app is…because your phone is different right? the colour is different, then the camera is different; those are things that of [affects] the confidence level of the application.* [SK04; 33-year-old Chinese mother of 1]

*Hmm, because I think it depends on the lighting, so that’s why I don’t use [it].* [BM10; 38-year-old Chinese mother of 1]

They knew that bilirubin levels measured using a transcutaneous bilirubinometer and blood test differed. Thus, they anticipated a similar difference in the readings from the HSMA and the actual jaundice level. If there was a difference, they wanted to know the difference in the accuracy of the app reading compared to the actual jaundice level, as a “small difference” could change how the doctor will manage the child.

*I don’t think it’s comparable because even when we do the first round of checks, using that meter [transcutaneous bilirubinometer] it can also differ from the blood test results. I don’t think it will be totally comparable.* [BM15; 31-year-old Chinese mother of 2]

*If let’s say it’s high, then I still need phototherapy [and] I will go down [to the clinic]. if it [threshold for phototherapy] is supposed to be below 200, then my levels were like 190, then to me, it seems ok, but is it accurate? Is the app accurate? What if it’s 200…what if there’s a 10% discrepancy.* [SK04; 33-year-old Chinese mother of 1]

Nevertheless, mothers trusted that the app would have been trialed and tested before being rolled out. Their trust in the service provider influenced their intention to use HSMAs in the future.

*I assume that if you have rolled it out you have done a lot of pilot tests and ascertain that the app is accurate.* [BM04; 33-year-old Chinese mother of 1]

Mothers suggested that health care professionals demonstrate and calibrate the HSMA on their devices in comparison with the current transcutaneous and serum bilirubin measurement. This would inform and reassure them that the app is accurate and reliable in assessing the jaundice level.

*If there’s a level of comparison, the phone app and the actual scanning, then you can see the level of discrepancy, let’s say if it’s 1% then it’s like ok, it’s quite accurate, not so bad.* [SK04; 33-yeary-old Chinese mother of 1]

### Usability

The simple interface of the app and the clear demonstration shown in the video facilitated the perceived ease of use. However, mothers were concerned about the procedural steps in using HSMAs and the app’s instructions being restricted to the English language, which may hinder its use. Mothers alluded to improving its ease of use by receiving adequate guidance from health care professionals and pairing teleconsultations with it.

#### Easy to Use

Most mothers perceived HSMAs as easy to use, as the video demonstrated the use of the app in simple, clear steps.

*Yes****,*** *it is quite easy. The steps are all very clear.* [BM13; 30-year-old Chinese mother of 1]

#### Instructions Restricted to the English Language

A mother worried that if the video instructions were only available in English, it might restrict the app’s use to a specific population. She suggested providing the instructions in multiple languages.

*I guess for most people, should be quite straight-forward but maybe because the video is only in English, I don’t know if it’s easy for everybody to understand the instructions…if it can be provided in different languages it would be helpful.* [SK01; 38-year-old Chinese mother of 1]

#### Higher Trust in Health Care Professionals

A few mothers doubted that they could use the HSMA correctly. They preferred having a health care professional evaluate their child for NNJ; they dreaded a delay in their child’s treatment if they misused the app.

*I think we would feel better, we will feel secure if we could actually come down [to the clinic] and get the nurse and the doctor to check properly. At the same time, you don’t know whether you’re doing it right and you don’t want to do, not knowing that’s the wrong way of doing and if there’s any appropriate action that you need to take and you are unable to…you go to the hospital/clinic also, I don’t know if I’m doing it right or wrong you know. I think it’s safer for a mother, anything to do with the child*…*go to the clinic. Let the doctors do whatever they need to do.* [BM01; 28-year-old Sikh mother of 1]

#### Guidance for App Use and the Management of Jaundice

Instruction by health care professionals on the use of the HSMA at the initial visit was favored by the mothers, as this provides an opportunity for them to clarify their doubts. Mothers suggested including a training manual in the app, a list of frequently asked questions with answers, and a hotline to assist them to resolve any problems.

*…they [the nurse or the doctor] can demonstrate to me and then if I’m not too sure about anything they can show me. I think a first demonstration by them will be fine*. [BM01; 28-year-old Sikh mother of 1]

*…if there’s a standard setting to take the photo then that can be communicated to mums...that could be helpful.* [SK04; 33-year-old Chinese mother of 1]

*As long as I’ve been taught how to do it, like troubleshoot with the FAQ, or hotline I could call, then I think that would be good.* [SK05; 36-year-old Chinese mother of 2]

Mothers wanted the app to include a guide to provide an action plan associated with the readings and clinical signs, as they perceived a paucity of web-based references to the measurements.

*After you know the result, you need to [inform] parents what to do next. If it is this colour, can we still monitor for the next two days? or need to call doctor or need to go to hospital?* [BM07; 34-year-old Chinese mother of 1]

*Actually, there was no online gauge to what’s the level that it should be…only when we went to the hospital, then we found out like…physical signs to look at. I think that could be part of the app as well.* [SK04; 33-yeary-old Chinese mother of 1]

#### Incorporation Into Teleconsultations

Mothers proposed pairing the use of HSMAs with teleconsultations. They felt that using an app during teleconsultations would allow them to avoid bringing their child to the clinic and help demonstrate an objective NNJ assessment to the attending health care professional in real time.

*I will use [teleconsultation]. I am very scared to bring the baby out.* [BM07; 34-yeary-old Chinese mother of 1; when asked about using teleconsultations to monitor for NNJ]

## Discussion

### Principal Findings

This qualitative research study highlights the mothers’ perceived usefulness of HSMAs. Mothers valued HSMAs for the convenience and objectivity when compared to visual inspection but had reservations about the accuracy. The app’s user-friendly interface as shown in the demonstration video put parents at ease, although concerns were raised with regard to the instructions being restricted to the English language. Mothers suggested that guidance from health care professionals, validation of its accuracy, and integration with teleconsultations could boost the usability of the app.

Mothers viewed self-monitoring of NNJ using an mHealth app at home as a convenient alternative to multiple primary care clinic check-ups. Similarly, in an earlier study by Yan et al [19], more than half of mothers rated a smartphone app as convenient for monitoring their child for NNJ remotely. Remote access to an NNJ-monitoring service can overcome multiple inconveniences such as rushed clinic visits, long waits in the clinic, the unavailability of transportation, long travel distances, and the cost of traveling [6]. An earlier study also demonstrated that remote monitoring of NNJ reduced the number of outpatient visits without increasing the risk of readmission and severe neonatal hyperbilirubinemia [20].

In addition, pairing HSMAs with telemedicine or video consultations, as the mothers suggested, would be favored by those who prefer remote consultations in the convenience of their homes. Telemedicine can also reduce nosocomial infection or other adverse health effects in neonates and improves mothers’ mental health [19,21]. The use of teleconsultation in a multisite neonatal follow-up program improved the consultation rates with a show rate of 95% [22]. This pairing will also improve mothers’ confidence in using the app when NNJ is monitored objectively using HSMAs along with guidance from a health care professional via video consultation.

Mothers indicated that the app was easy to use based on the video demonstration. In an earlier study, Jin et al [23] highlighted the ease of use as a significant public consideration in the adoption of mHealth apps. Most mothers were familiar with phone-based app because of Singapore’s high smartphone penetration; the current smartphone penetration in 2023 is 95.4%, which is expected to increase to 99.4% by 2028 [24]. Furthermore, the Singapore government encourages harnessing technology to monitor and manage health conditions [25]. These enablers support the uptake of validated mHealth apps in health care delivery.

The mothers’ concerns about the accuracy of HSMAs and the inherent reliance of such apps on good image quality and standardized lighting are valid. Earlier studies on NNJ-monitoring mHealth apps demonstrated discrepancies and false positives, which could potentially be contributed to heterogeneous lighting conditions and photo quality [10,26]. Thus, for imaged-based mHealth apps, optimal image quality and standardized lighting conditions are crucial factors to be addressed to achieve reliable results. These concerns about image quality and lighting can be addressed through the app’s built-in capability to reject low-quality images [17,26]. Additionally, the use of a color calibration card compensates for variations in light intensity [17,26]. A novel mHealth app that overcomes these limitations has been developed by the lead authors and will undergo clinical validation; this validation study will include a quantitative component to complement the qualitative findings.

Mothers in this study emphasized the need to conduct training before using the app; this would improve the app’s usability. Lewis and Wyatt [27] reported inadequate user training of mHealth apps, resulting in inappropriate app use and users’ failure to detect app malfunction or accurately diagnose the health condition. As the mothers in this study perceived, a live demonstration of the app by health care professionals is more valuable than an instructional video. Prompt user guidance on monitoring and advice on individualized actionable management of NNJ would encourage the use of mHealth apps appropriately [28]. Providing the relevant information for specific health conditions and corresponding feedback for tests enhances user satisfaction [28]. Thus, user training is pivotal to ensure the app’s correct use and reduce possible risks to patient safety.

Another barrier to the HSMA’s usability was its sole dependency on the English language to provide instructions to users. Communicating with users in languages they can understand is one of the most pertinent factors influencing an app’s utility [29]. Nevertheless, English is the official language for education and work in Singapore. The country has a high literacy rate of 97.1%, and almost half of the population (48.3%) use English as their main language at home [30]. However, a minor group of residents or emigrants from overseas countries such as China, South Asia, Asia-Pacific, and other regions may not be as proficient in English as native Singaporeans and could deter them from using an English-based app. As such, incorporating more languages in the app could extend the reach to more users and will be developed at a later stage.

### Strength and Limitations

This is likely the first study exploring Asian mothers’ attitudes toward using a smartphone app for self-monitoring of NNJ in a technologically advanced community. Using qualitative research to explore the attitude among mothers provided in-depth knowledge of their needs, barriers, and intent to use such an mHealth app.

The study has several limitations. First, the study describes the perceived usefulness of the NNJ-monitoring mHealth app based on a video demonstration instead of the actual use of the app; thus, the usefulness of the app in the real world may differ. Nevertheless, understanding the end user’s perspective is essential in designing and developing the app. The data gathered on the mother’s needs of such an mHealth app from this study are sufficient to guide the developers and health care professionals to design and test the prototype on screening for NNJ remotely. Second, the mothers in this study were highly educated, restricting the study’s generalizability. A more diverse range of participants in terms of education levels and socioeconomic backgrounds could enhance the generalizability of the findings. Nevertheless, the Singapore census shows that 80% to 90% of people aged 25-50 years (the usual age group of mothers) have completed postsecondary or higher education [30]. This is consistent with most of the tertiary-educated mothers in this study.

### Conclusion

Mothers valued the convenience and objectivity of HSMAs and perceived them as easy to use. The app’s exclusive use of the English language was raised as potential barriers to its usability and utility. To increase its successful adoption, multiple languages, proof of accuracy, and resources to guide users would be incorporated in the next phase of the study. Combining mHealth apps and teleconsultations to monitor for NNJ remotely represents an accessible and pragmatic care delivery model.

## Supplementary material

10.2196/53291 Checklist 1 Consolidated Criteria for Reporting Qualitative Research (COREQ) checklist.

## Abbreviations

BM: Bukit Merah
COREQ: Consolidated Criteria for Reporting Qualitative Research
HSMA: hyperbilirubinemia-screening mobile health app
mHealth: mobile health
NNJ: neonatal jaundice
SK: Sengkang
